# Supplementary material for: Use of steric blocking antisense oligonucleotides for the targeted inhibition of junction containing precursor microRNAs
Source: bioRxiv. 2024 Apr 8:2024.04.08.588531. Preprint. [Version 1] doi: 10.1101/2024.04.08.588531 (PMC11030329; doi:10.1101/2024.04.08.588531)
Supplement: Supplement 1 [file media-1.pdf]

SUPPLEMENTARY DATA

Use of steric blocking antisense oligonucleotides for the targeted inhibition of junction containing precursor microRNAs

AUTHORS

Sicong Ma[1], Samantha A. Howden[3], and Sarah C. Keane[1,2]*

[1]Program in Biophysics, University of Michigan, Ann Arbor, MI 48109, USA

[2]Department of Chemistry, University of Michigan, Ann Arbor, MI 48109, USA

[3]College of LSA, University of Michigan, Ann Arbor, MI 48109, USA

* To whom correspondence should be addressed. Tel: 1-734-763-2332 ; Email: sckeane@umich.edu

Table S1. Overlap extension PCR primers for generation of pre-miRNA templates.

Construct	Primer name	5'-sequence-3'[a]
HH-pre-miR-21	HH-pre-miR-21-1F	TAATACGACTCACTATAGGCTCG
	HH-pre-miR-21-2R	ACCCTGATGGTGTCTGAAAAACGTACCCTGATGGTGTACGAGCCTATAGTGAGTCGTAT
	HH-pre-miR-21-3F	TTCAGACACCATCAGGGTCTGCTGATAAGCTACTGATGAGTCCGTGAGG
	HH-pre-miR-21-4R	CTAGACGGTACCGGGTACCGTTTCGTCCTCACGGACTCATCAGTA
	HH-pre-miR-21-5F	ACCCGGTACCGTCTAGCTTATCAGACTGATGTTGACTGTTGAATCTCATGGCAACACCAG
	HH-pre-miR-21-6R	mGmACAGCCCATCGACTGGTGTTGCCATGAGATT
pre-miR-let7c	pre-miR-let7c-nat-1F	CCGGAATTCTAATACGACTCACTATAGGGCTCGTACACCATCAGGGTACGTTTTTCA
	pre-miR-let7c-nat-2R	GCAGACCCTGATGGTGTCTGAAAAACGTACCCTGATGGTGTACGAGCC
	pre-miR-let7c-nat-3F	ACACCATCAGGGTCTGCTACTACCTCACTGATGAGTCCGTGAGGACGAA
	pre-miR-let7c-nat-4R	CCTCAGACGGTACCGGGTACCGTTTCGTCCTCACGGACTCAT
	pre-miR-let7c-nat-5F	CCGGTACCGTCTGAGGTAGTAGGTTGTATGGTTTAGAGTTACACCCTGGGAGTTAACTGT
	pre-miR-let7c-nat-6R	CCGTCGCGGATCCGGAAAGCTAGAAGGTTGTACAGTTAACTCCCAGGGTGTAA
HH-pre-miR-144	HH-pre-144-1F	TAATACGACTCACTATAGGCTCG
	HH-pre-144-2R	ACCCTGATGGTGTCTGAAAAACGTACCCTGATGGTGTACGAGCCTATAGTGAGTCGTATT
	HH-pre-144-3F	TTCAGACACCATCAGGGTCTGTGATGATATCCCTGATGAGTCCGTG
	HH-pre-144-4R	CGACGGTACCGGGTACCGTTTCGTCCTCACGGACTCATCAGGGA
	HH-pre-144-5F	ACCCGGTACCGTCGGATATCATCATATACTGTAAGTTTGCGATGAGACACTAC
	HH-pre-144-6R	mAmGTACATCATCTATACTGTAGTGTCTCATCGCAAACTT
GG-pre-miR-19a	GG-pre-miR19a-F	TAATACGACTCACTATAGGTTTTGCATAGTTGCACTACAAG
	GG-pre-miR19a-R	mUmCAGTTTTGCATAGATTTGCACAACTACATTCTTCTTGTAGTGCAACTATGCAAAACC
Pre-miR-143	pre-miR143-F	TAATACGACTCACTATAGGTGCAGTGCTGCATCTCTGGTCAGTT
	pre-miR143-R	mGmAGCTACAGTGCTTCATCTCAGACTCCCAACTGACCAGAGATGCAGCACTGCACCT

[a] m denotes 2′-*O*-Me modification of the primer.

Table S2. Amplification primers for template.

Amplification primer	5'-sequence-3'[a]	application
UNIV-pUC19_E105	TCTTCGCTATTACGCCAGCTGGCGAAA	Forward primer for amplification of DNA template for HH-pre-let-7c, HH-pre-miR-31 from plasmid
HDV-AMP-R	mUmAATGTGAGAATTGGCTACGTTGAAACAACGCATTACCG	Reverse primer for amplification of DNA template for HH-pre-miR-31-HDV from plasmid
HH-pre-let7c-AMP-R	mGmGAAAGCTAGAAGGTTGTACAGTTAACTCCCAGGG	Reverse primer for amplification of DNA template for HH-pre-let-7c from plasmid

[a] m denotes 2′-*O*-Me modification of the primer.

Table S3. RNA sequences.

RNA name	5'-sequence-3'[a]
pre-miR-31	AGGCAAGAUGCUGGCAUAGCUGUUGAACUGGGAACCUGCUAUGCCAACAUAUUGCCAU
pre-miR-21	UAGCUUAUCAGACUGAUGUUGACUGUUGAAUCUCAUGGCAACACCAGUCGAUGGGCUGU
pre-let-7c	UGAGGUAGUAGGUUGUAUGGUUUAGAGUUACACCCUGGGAGUUAACUGUACAACCUUCUAGCUUUCC
pre-miR-144	GGAUAUCAUCAUAUACUGUAAGUUUGCGAUGAGACACUACAGUAUAGAUGAUGUACU
GG-pre-miR-19a	gGUUUUGCAUAGUUGCACUACAAGAAGAAUGUAGUUGUGCAAAUCUAUGCAAAACUGA
pre-miR-143	GGUGCAGUGCUGCAUCUCUGGUCAGUUGGGAGUCUGAGAUGAAGCACUGUAGCUC

[a]Non-native residues are in lowercase.

Table S4. Antisense oligo sequences.

Antisense oligo	primer names	5'-sequence-3'[a]
Anti-miR31-A1	anti-miR31-D3	CAGGTTCCCAGTTCAACAG
Anti-miR31-A2	anti-miR31-loop-D	AGGTTCCCAGTTCAACA
Anti-miR31-A3	anti-miR31-D1	GGTTCCCAGTTCAAC
Anti-miR31-A4	anti-miR31-D2	GTTCCCAGTTCAAC
Anti-miR31-A4-LNA	anti-miR31-D2-LNA	+G*+T*+T*C*+C*C*A*+G*T*+T*C*+A*+A*+C
Anti-miR144-A1	Anti-miR144-D3	AGTGTCTCATCGCAAACTT
Anti-miR144-A2	Anti-miR144-loop-D	GTGTCTCATCGCAAACT
Anti-miR144-A3	Anti-miR144-D1	GTGTCTCATCGCAAA
Anti-miR144-A4	Anti-miR144-D2	GTCTCATCGCAAAC
Anti-miR144-A4-LNA	anti-miR144-D2-LNA	+G*+T*+C*T*+C*A*T*+C*G*+C*A*+A*+A*+C
Anti-miR19a-A2	Anti-miR19a-loop-D	AACTACATTCTTCTTGTA
Anti-miR143-A4	Anti-miR143-D2	GACTCCCAACTGACC
Anti-let-7c-A1	pre-let-7c-AP-ASO	TCCCAGGGTGTAAC
ASO (-) control	anti-miR-31-locked	+C*C*+G*T*T*+C*+T*A*C*+G*A*+C*C*+G*+T
Anti-miR-31 (+) control	Horizon Discovery	Catalog ID: IH-300507-06
Anti-miR-144 (+) control	Horizon Discovery	Catalog ID: IH-300612-06

[a]'*' indicates phosphorothioate backbone, '+' indicates locked nucleic acid.

Table S5. Templates for generating pmirGLO plasmid inserts.

Construct	Primer name	5'-sequence-3'
pmirGLO miR31	pmirGLO-miR-31-F	AAACTAGCGGCCGCTAGTAGCTATGCCAGCATCTTGCCTT
	pmirGLO-miR-31-R	CTAGAAGGCAAGATGCTGGCATAGCTACTAGCGGCCGCTAGTTT
pmirGLO miR144	pmirGLO-miR144-N-F	GCGTTTAAACTAGCGGCCGCTAGTAGTACATCATCTATACTGTATCTAGAGC
	pmirGLO-miR144-N-R	GCTCTAGATACAGTATAGATGATGTACTACTAGCGGCCGCTAGTTTAAACGC

Table S6. Overlap extension PCR primers for generation of pCMV miR31 plasmid insert.

Construct	Primer name	5'-sequence-3'
pCMV miR31	cmv-miR31-1F	GCAGATCTAGTCATAGTATTCTCCTGTAACTTGGAACTG GAGAGGAGGCAAGATGCT
	cmv-miR31-2R	GGCATAGCAGGTTCCCAGTTCAACAGCTATGCCAGCATC TTGCCTCCTCT
	cmv-miR31-3F	TGGGAACCTGCTATGCCAACATATTGCCATCTTTCCTGTC TGACAGCAGCCATGGCCAC
	cmv-miR31-4R	GCCTCGAGGCATGCAGGTGGCCATGGCTGCTGTCAGACA GGAAA

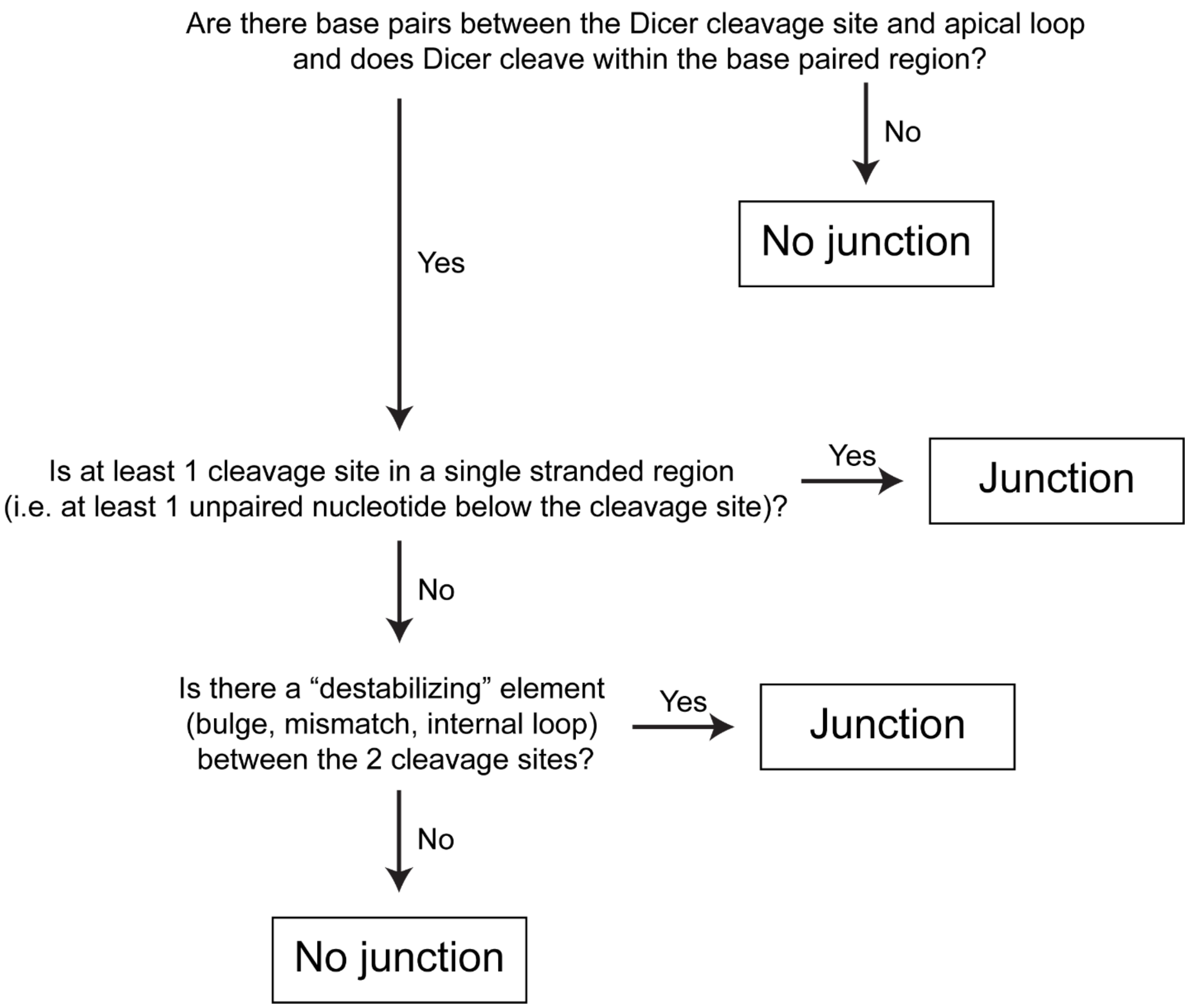

Figure S1 Flowchart of junction region-containing pre-miRNAs determination.

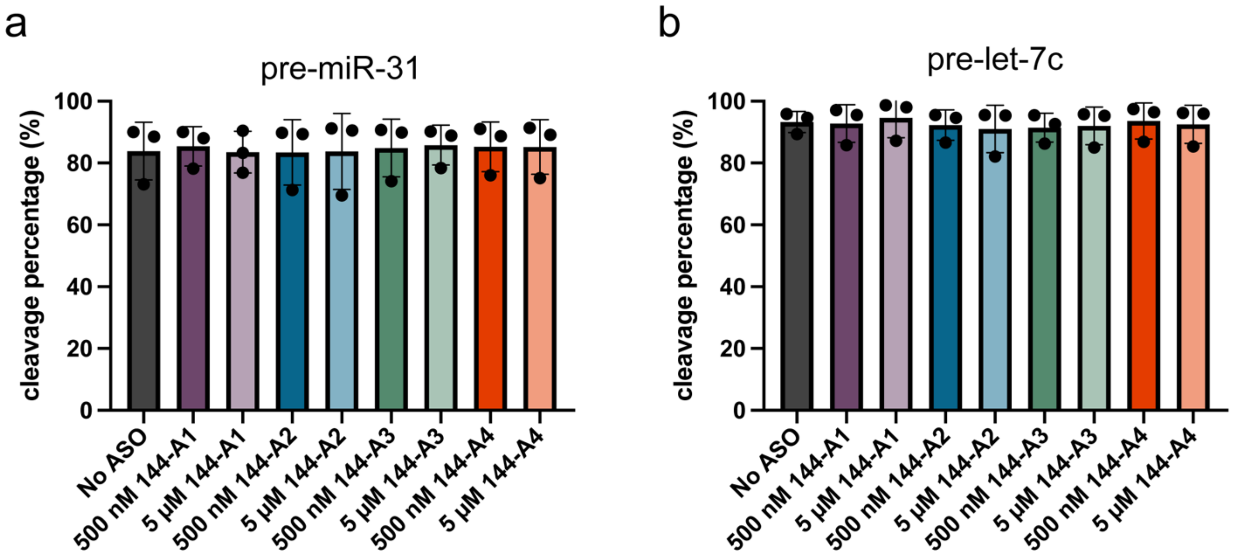

Figure S2. Anti-miR144 ASO are specific and do not affect the processing of pre-miR-31 or pre-let-7c. **a)** Dicer-TRBP processing of pre-miR-31 in the presence and absence of different anti-miR144 ASOs. **b)** Dicer-TRBP processing of pre-let-7c in the presence and absence of different anti-miR144 ASOs. No significant differences were identified from an ordinary one-way ANOVA Tukey analysis.

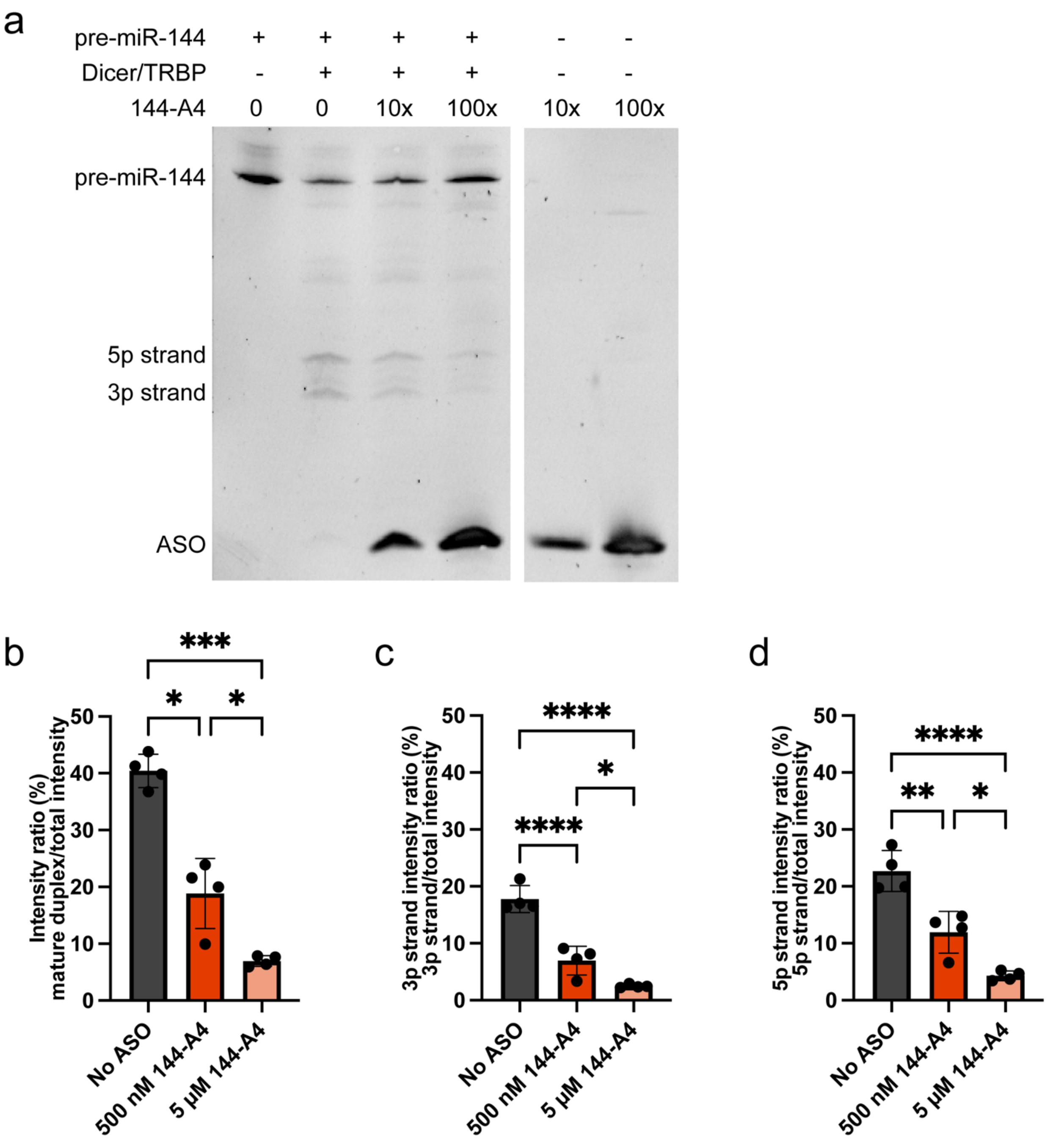

Figure S3. ASO 144-A4 inhibits Dicer-TRBP processing of pre-miR-144. **a)** Representative pre-miR-144 processing assay, visualized by SYBR gold staining. **b-d)** Quantification of pre-miR-144 processing products. **** $p<0.0001$, ***$p<0.001$, **$p<0.01$, and *$p<0.05$ from an ordinary one-way ANOVA Tukey analysis.

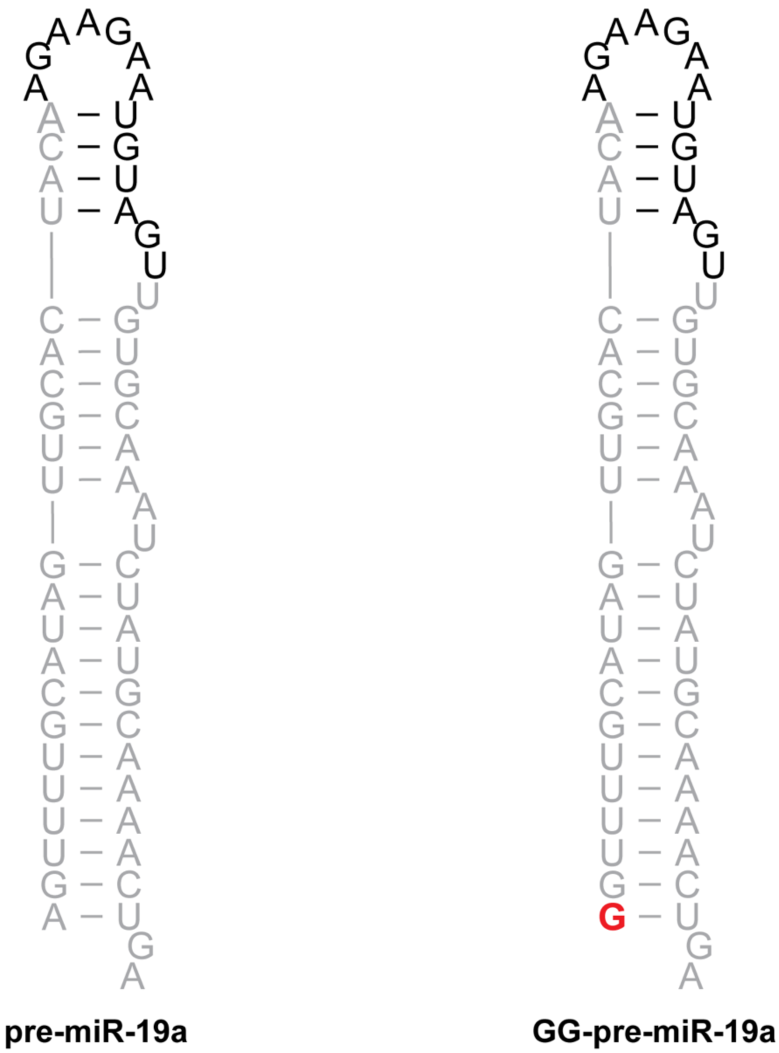

Figure S4. Secondary structure of pre-miR-19a and GG-pre-miR-19a.

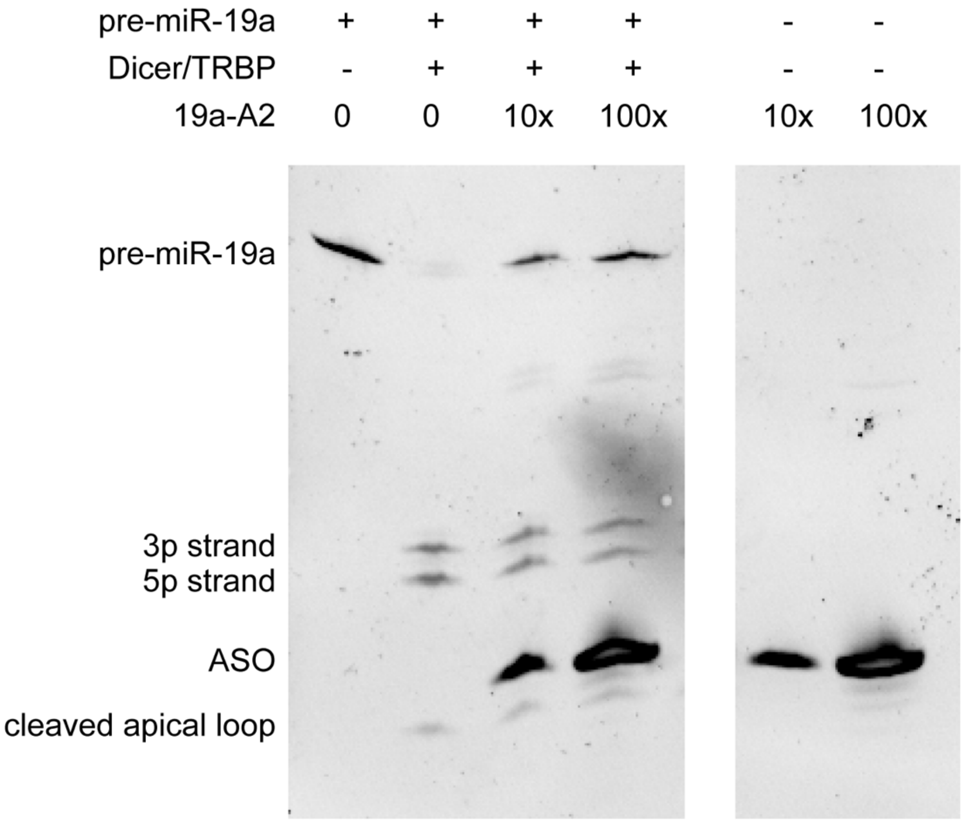

Figure S5. Representative GG-pre-miR-19a processing assay, visualized by SYBR gold staining.

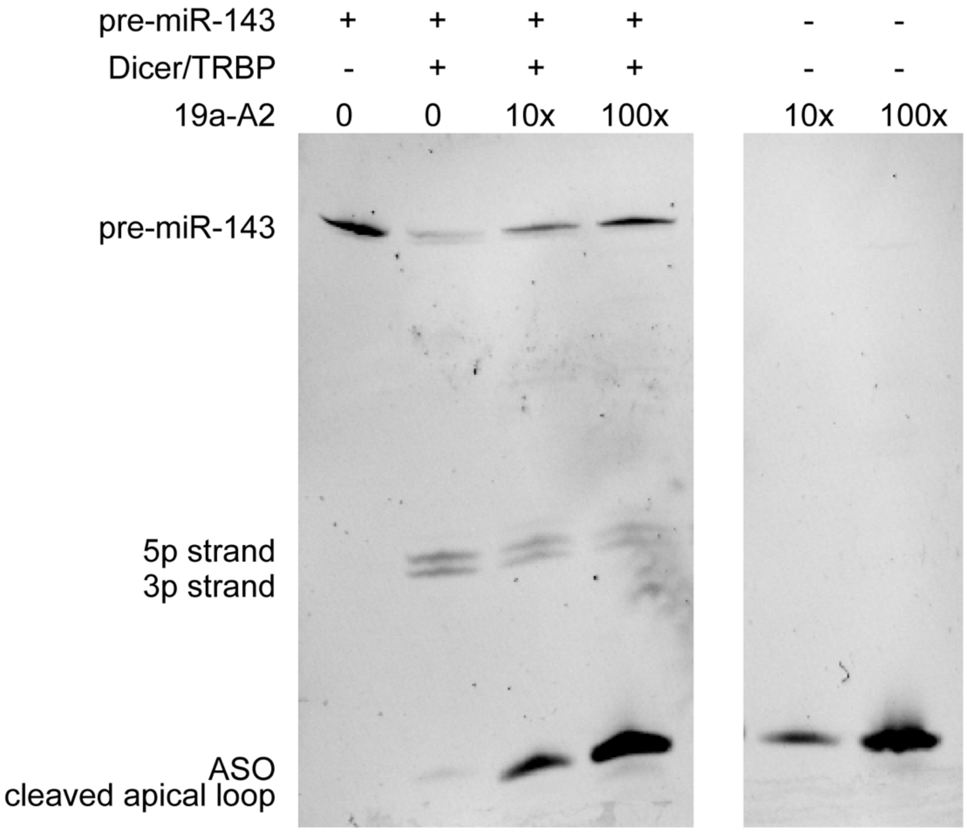

Figure S6. Representative pre-miR-143 processing assay, visualized by SYBR gold staining.

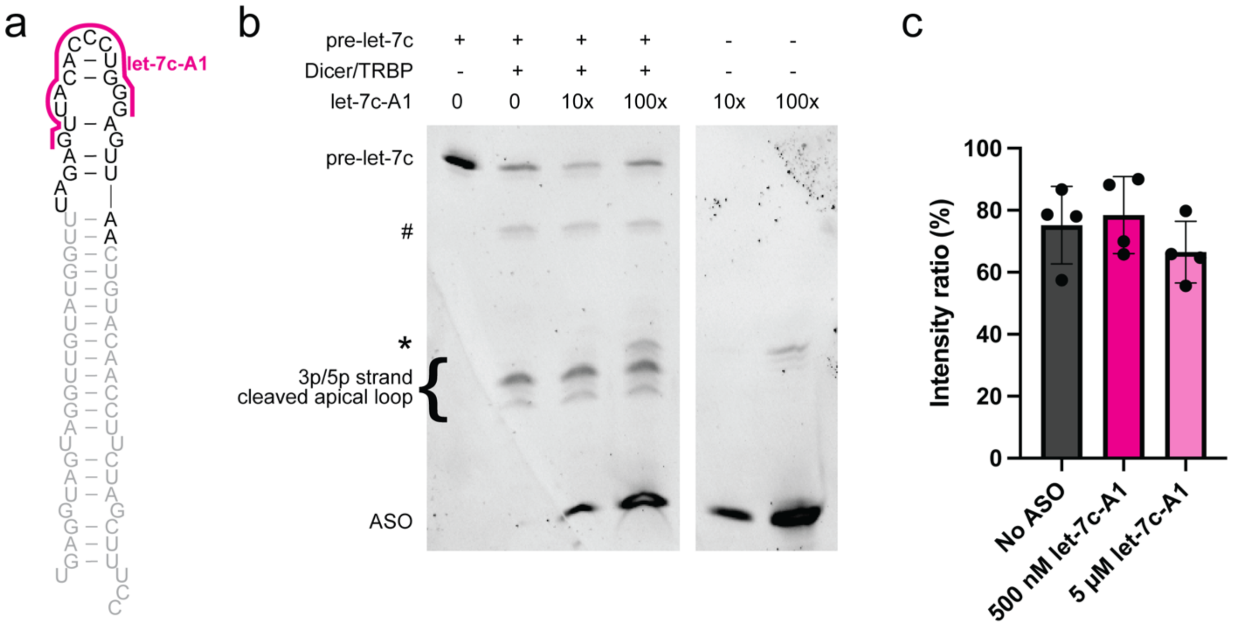

Figure S7. a) Predicted secondary structure of pre-let-7c. The mature let-7c duplex sequence is gray and cleaved off apical loop is black. **b**) Representative pre-let-7c processing assay, visualized by SYBR gold staining. # indicates single processed pre-let-7c product, * indicates an impurity from the anti-let-7c-A1 ASO. **c)** The intensity ratio of mature let-7c duplexes is not affected as let-7c-A1 concentration increases in the reaction.
